# ASSOCIATION BETWEEN TROUBLE SLEEPING AND ORAL CONDITIONS AMONG SCHOOLCHILDREN

**DOI:** 10.1590/1984-0462/2021/39/2019342

**Published:** 2020-09-25

**Authors:** Juliana Moro, Pablo Santos, Angela Giacomin, Mariane Cardoso, Michele Bolan

**Affiliations:** a Universidade Federal de Santa Catarina, Florianópolis, SC, Brazil.

**Keywords:** Dental caries, Child, Sleep, Cárie dentária, Crianças, Sono

## Abstract

**Objective:**

To investigate the prevalence of self-reported trouble sleeping due to dental problems and its association with oral conditions in schoolchildren.

**Methods:**

This is a cross-sectional study carried out with a representative sample of 1,589 schoolchildren aged 8-10 years enrolled in public schools from Florianópolis, Santa Catarina, Brazil. Non-clinical data included a questionnaire about socioeconomic indicators answered by parents/guardians. Children were questioned about whether they had trouble sleeping due to dental problems and about previous history of toothache. Clinical oral examinations were performed to evaluate dental caries - Decayed, Missing, and Filled Teeth Index (DMFT/dmft index) and its clinical consequences [PUFA/pufa index: considering the presence of pulpal involvement (P/p); ulceration of tissues due to tooth fragments from decayed crowns (U/u); fistula (F/f); and abscesses (A/a), and traumatic dental injuries (TDI)]. We conducted a descriptive analysis and used adjusted logistic regression models (p<0.05; 95%CI).

**Results:**

The prevalence of trouble sleeping due to dental problems was 28%. Children with untreated dental caries (OR 1.32; 95%CI 1.05-1.67) and clinical consequences from the PUFA/pufa index (OR 1.89; 95%CI 1.45-2.46) had higher chances of reporting trouble sleeping due to dental problems.

**Conclusions:**

Approximately one-third of the children declared having trouble sleeping due to dental problems. Untreated dental caries and its clinical consequences were associated with self-reported trouble sleeping due to dental problems in schoolchildren.

## RESUMO

**Objetivo:**

Investigar a prevalência de problemas para dormir por motivos dentários autorrelatado e sua associação com condições orais adversas em escolares.

**Métodos:**

Estudo transversal com amostra representativa de 1589 escolares de 8 a 10 anos matriculados em escolas públicas de Florianópolis, Brasil. Os dados não clínicos incluíram um questionário sobre indicadores socioeconômicos respondidos pelos pais/cuidadores. As crianças foram questionadas se tinham problemas para dormir por motivos dentários e sobre episódios anteriores de dor de dente. Foram realizados exames clínicos orais para avaliar: cárie dentária - Índice de Dentes Cariados, Perdidos e Obturados (índice CPO-D/ceo-d) - e suas consequências clínicas (índice PUFA/pufa - presença de envolvimento pulpar (P/p); ulceração (U/u); fístula (F/f); e abscessos (A/a) e traumatismo dental (TD). Foram realizados análise descritiva e modelos ajustados de regressão logística (p<0,05; IC95%).

**Resultados:**

A prevalência de problemas para dormir por motivos dentários foi de 28%. Crianças com cárie dentária não tratada (OR 1,32; IC95% 1,05-1,67) e presença de índice PUFA/pufa (OR 1,89; IC95% 1,45-2,46) apresentaram maiores chances de relatar problemas para dormir devido a razões dentárias.

**Conclusões:**

Aproximadamente, um terço das crianças apresentou problemas para dormir devido a razões dentárias. Cárie dentária não tratada e suas consequências clínicas foram associadas a problemas autorrelatados para dormir por motivos dentários em escolares.

## INTRODUCTION

Sleep is an important component to achieve a proper development as well as maintain the mental and physical health.^1^ However, research from the American National Sleep Foundation showed that 27% of school-aged children experience inadequate sleep, and 45% of adolescents sleep less than 8 hours per night.^2^

Trouble sleeping is often observed in childhood. Researchers estimate that 31% of children aged 6 to 13 years develop trouble initiating and maintaining sleep.^3^ Sleep deprivation is known to have a negative impact on quality of life, learning, memory, and school performance of children.^1^ ^,^ ^4^ Children with inadequate sleep also tend to experience more mood changes and emotional insecurity, jeopardizing social relations.^5^

Studies on the causes of sleep pattern disturbance in schoolchildren are essential, as they may help parents and guardians to establish good health habits that promote adequate sleep in children. Several factors may influence sleep duration, such as chronic pain and other medical conditions.^6^ Dental caries is one of the most prevalent diseases in childhood and, if left untreated, can generate pain, discomfort, and infection, jeopardizing the child's quality of life and daily activities, including difficulty initiating and maintaining sleep.^7^ ^,^ ^8^

In this sense, evaluating oral conditions as potential predictors of trouble sleeping may be important because of the negative effects they can have on children. Although there are studies on the impact of oral conditions on activities of daily living for children,^7^ ^,^ ^8^ ^,^ ^9^ ^,^ ^10^ ^,^ ^11^ ^,^ ^12^ few have evaluated if the caries experience and its clinical consequences are associated with trouble sleeping. Therefore, the present study aimed to investigate the prevalence of self-reported trouble sleeping due to dental problems and whether it has an association with oral conditions in schoolchildren aged 8 to 10 years.

## METHOD

The Human Research Ethics Committee of Universidade Federal de Santa Catarina (process No. 902,633/2015) and the Municipal Secretariat of Education approved this study. All children agreed to participate by signing an agreement form, while their parents or guardian signed an informed consent form.

The present cross-sectional study was carried out with children aged 8 to 10 years from public schools of Florianópolis, Santa Catarina, Brazil. Data were collected from May to December 2015. Florianópolis is a city from Southern Brazil that has 36 public schools, which included 16,234 elementary school children in 2015.

Sample size calculation was based on a prevalence of trouble sleeping of 31%.^3^ We adopted a 95% confidence interval (95%CI), 5% standard error, and 80% power. The minimum sample size required was 329 participants in each age stratum, resulting in an initial sample of 987 children. A 1.5 correction factor was used to compensate for the clustering effect, and 10% was added to compensate for possible losses. Therefore, the final estimated sample was 1,629 schoolchildren.

The subjects were randomly selected using a two-stage sampling method. The first stage was the randomization of public schools, and the second was the randomization of the classrooms. The cluster sampling was performed with the draw of public schools, maintaining the proportionality between the number of schools and 2^nd^- to 5^th^-grade students in the districts, followed by the draw of classrooms, also respecting the proportion of the number students. Each stage involved a simple draw with the aid of an online randomizer tool (randomizer.org).

Eighteen schools were selected to compose the calculated sample. After contacting the supervisors, two schools refused to participate. A new draw was carried out to complement the sample. All 8- to 10-year-old children enrolled in the selected classrooms were invited to participate.

The sample included children aged 8 to 10 years, regularly enrolled in a public school, and accompanied by a Brazilian Portuguese-speaking guardian. Children with diseases that affected the central nervous system and those who were taking medications that could interfere with the central nervous system were excluded from the study due to the possible disruption to the sleep pattern.

The calibration exercise comprised two phases. Initial theoretical training was conducted to present and discuss the criteria for the diagnosis of dental caries, PUFA/pufa index, and traumatic dental injury (TDI). Subsequently, the calibration between the four examiners and the gold standard, a specialist in pediatric dentistry, was carried out with the oral examination of 20 children aged 8 to 10 years. The children were examined at two different times, with a 7- to 14-day interval between them. The intra-rater Cohen’s *Kappa* coefficient for dental caries, PUFA/pufa index, and TDI were >0.75, and inter-rater values were >0.70.

Following the calibration exercise, a pilot study was conducted in a random public school with 33 children and their guardians to test the study methodology reproducibility. The pilot study involved the administration of two questions about trouble sleeping and history of toothache (answered by the children) and a questionnaire of socioeconomic indicators (answered by the guardians). Furthermore, a clinical oral examination was conducted to verify the criteria reliability and diagnostic accuracy. No methodological changes were necessary since the instruments were considered appropriate for the study purpose. The children included in the pilot study were excluded from the main study.

Information about trouble sleeping was collected through a question directed to the children: “Have you had trouble sleeping at night due to dental problems?” The answers were dichotomized as yes or no. The history of toothache was obtained by a single question: "Have you ever had a toothache?", also dichotomized as yes or no. The questions were based on the Child Perceptions Questionnaire (CPQ_8-10_).^13^ Possible answers and scores varied as follows: “Never”=0; “Once or twice”=1; “Sometimes”=2; “Often”=3; “Every day or almost every day”=4. The variables were dichotomized as no trouble sleeping/toothache (answer 1) and trouble sleeping/toothache (answers 2, 3, 4, or 5).

The socioeconomic and demographic characteristics of the family, as well as the age and gender of children, were obtained from a questionnaire answered by the parents/guardians. The guardians' level of schooling was dichotomized as ≤8 years and >8 years of study, and household income was divided into ≤2 Brazilian Minimum Wages (BMW) and >2 BMW.^14^ The BMW was approximately US$ 208 in 2015.

Four calibrated examiners collected the clinical data in the school environment. Children were seated in a school chair, and the examiners performed the visual inspection of the oral cavity with the aid of an artificial light (Petzl Zoom headlamp; Petzl America, Clearfield, UT, USA) and no. 5 mouth mirrors (PRISMA, São Paulo, SP, Brazil).

The DMFT/dmft index was used to assess the caries experience in deciduous and permanent teeth.^15^ The Caries Experience variable (DMFT/dmft) expressed the total number of teeth (T/t) that were decayed (D/d), missing due to dental caries (M/m), or filled (F/f). The statistical analysis considered a child "caries-free" when the DMFT/dmft index was 0. Untreated dental caries was determined to isolate the “decayed” (D) component of the DMFT/dmft index. We used the PUFA/pufa index^16^ to assess the clinical consequences of untreated caries lesions, considering the presence of pulpal involvement (P/p); ulceration of tissues due to tooth fragments from decayed crowns (U/u); fistula (F/f); and abscesses (A/a). The PUFA/pufa index was dichotomized as yes (≥ 1 tooth with clinical consequences of dental caries) and no (no clinical consequences). Uppercase letters represent permanent teeth, and lowercase letters correspond to deciduous teeth in both the DMFT/dmft and PUFA/pufa indices.

Clinical information about TDI in the permanent anterior teeth was collected following the adapted classification by Andreasen et al.,^17^ including enamel fracture, enamel-dentine fracture, luxation, tooth discoloration, and avulsion. The TDI variable was dichotomized as yes and no.

Data analysis was performed using the STATA^®^ software (StataCorp LLC, Texas, EUA), version 13.0. We conducted descriptive and unadjusted analyses. Unadjusted and adjusted logistic regression models were used to analyze factors associated with trouble sleeping. The adjusted analysis employed the backward stepwise method. Adjustments were made for the independent variables presenting p≤0.20 in the unadjusted model. In the adjusted analysis, a p<0.05 was considered statistically significant. The statistical analysis considered the sample weights of children. We used the "svy: logistic" command for complex sample data. Results were expressed as *Odds Ratio* (OR) with a 95% confidence interval (95%CI).

## RESULTS

A total of 1,589 children participated in the present study, with a 97.54% response rate. The main reasons for non-participation (n=40) were unanswered questionnaires and missing data. Table 1 describes the variables. In the present study, the prevalence of schoolchildren who reported trouble sleeping due to dental problems was 28%. The sample consisted mainly of girls (58%), 9-year-olds (36%), children whose guardians had more than 8 years of schooling (69%), and with a household income of ≤2 BMW (51%). Regarding clinical variables, most schoolchildren had experienced dental caries (57%), whereas 43% had untreated dental caries, and 22% presented clinical consequences of untreated dental caries (pulpal involvement, ulceration, fistula, and/or abscess). Most children (52%) reported history of toothache. Only 11% of the children had TDI.

**Table 1:** Frequency of trouble sleeping and independent variables among schoolchildren. Florianópolis, Brazil (n=1,589).

	Frequency	
	n	%
Trouble sleeping		
No	1,140	72
Yes	449	28
Gender		
Male	673	42
Female	916	58
Age (years)		
8	533	33
9	570	36
10	486	31
Guardian’s schooling*		
>8 years	1,029	69
≤8 years	453	31
Household income		
≤2 BMW	804	51
>2 BMW	785	49
Caries experience		
No	686	43
Yes	903	57
Untreated dental caries		
No	898	57
Yes	691	43
PUFA/pufa^&^		
No	1,244	78
Yes	345	22
Toothache		
No	770	48
Yes	819	52
TDI**		
No	1,421	89
Yes	168	11
TOTAL	1,589	100

*Guardian’s schooling (n=1,482); **TDI: traumatic dental injury; ^&^PUFA/pufa: presence of pulpal involvement (P/p); ulceration of tissues due to tooth fragments from decayed crowns (U/u); fistula (F/f); and abscesses (A/a). BMW: Brazilian Minimum Wage.

Table 2 shows the results of the unadjusted and adjusted multivariate logistic regression models for possible predictors associated with trouble sleeping. After adjustment, children with untreated caries and clinical consequences from the PUFA/pufa presented, respectively, 89% (95%CI 1.45-2.46; p<0.01) and 32% (95%CI 1.05-1.67; p=0.02) more chance of reporting trouble sleeping when compared to those who had no untreated caries and no clinical consequences from the PUFA/pufa index. The other independent variables (gender, age, TDI, toothache, and caries experience) were not associated with trouble sleeping in schoolchildren.

**Table 2:** Unadjusted and adjusted multivariate logistic regression models for independent variables associated with trouble sleeping among schoolchildren. Florianópolis, Brazil (n=1,589).

	Trouble sleeping		Unadjusted		Adjusted	
	Yes	No	OR (95%CI)	p-value	OR (95%CI)	p-value
Gender						
Female	278 (30.3)	638 (69.7)	1			
Male	171 (25.4)	502 (74.6)	1.11 (0.88-1.40)	0.35	-	
Age (years)						
8	159 (29.8)	374 (70.2)	1			
9	163 (28.6)	407 (71.4)	0.94 (0.71-1.24)	0.67		
10	127 (26.1)	359 (73.9)	0.84 (0.63-1.13)	0.26	-	
Caries experience						
No	160 (23.3)	526 (76.7)	1			
Yes	289 (32)	614 (68)	0.86 (0.59-1.25)	0.44	-	
Untreated dental caries						
No	206 (22.9)	692 (77.1)	1		1	
Yes	243 (35.2)	448 (64.8)	1.47 (1.02-2.13)	0.04	1.32 (1.05-1.67)	0.02
PUFA/pufa index^&^						
No	313 (25.2)	931 (74.8)	1		1	
Yes	136 (39.4)	209 (60.6)	1.85 (1.24-2.42)	<0.01	1.89 (1.45-2.46)	<0.01
Toothache						
No	108 (14)	662 (86)	1			
Yes	341 (41.6)	478 (58.4)	1.01 (0.80-1.29)	0.85	-	
TDI*						
No	119 (10.4)	1,021 (89.6)	1			
Yes	400 (89)	49 (11)	1.10 (0.76-1.59)	0.61	-	

*TDI: traumatic dental injury; ^&^PUFA/pufa: presence of pulpal involvement (P/p); ulceration of tissues due to tooth fragments from decayed crowns (U/u); fistula (F/f); and abscesses (A/a); OR: *Odds Ratio*; 95%CI: 95% confidence interval.

## DISCUSSION

The present study aimed to investigate the prevalence of self-reported trouble sleeping due to dental problems and whether it has an association with oral conditions in schoolchildren. The main results revealed that untreated dental caries and its clinical consequences were associated with self-reported trouble sleeping in schoolchildren. Thus, oral health influences children's general health and quality of life. Sleep deprivation, especially in children and adolescents, may lead to low concentration and fatigue during the day and affect the child's mood and temperament.^1^ ^,^ ^4^ ^,^ ^5^ ^,^ ^18^ ^,^ ^19^ Insufficient sleep can compromise school performance, in addition to inducing higher rates of school absenteeism.^20^ ^,^ ^21^

In the present study, almost one-third of the children declared having trouble sleeping due to dental problems. Oral conditions had an impact on the sleep of 6.6% of children aged 2 to 5 years.^7^ Souza et al.^8^ showed that the incidence of sleep problems due to dental issues was 33% in 12-year-olds. The study by Lima et al.^10^ found a higher prevalence of sleep problems due to dental issues, with 72.8% in children aged 8 to 10 years. Given the importance of sleep for the development and growth of children,^1^ ^,^ ^22^ these results highlight the value of health promotion, as well as early diagnosis of oral conditions, since their treatment may help establish a better quality of childhood sleep.

Untreated dental caries in deciduous and/or permanent teeth led children to have greater chances of reporting trouble sleeping due to dental problems. This result corroborates the study by Souza et al.^8^ According to the authors, this finding can be explained by the greater discomfort and irritation generated by the condition, causing a negative impact on sleep. Lima et al.^10^ also identified that children aged 6 to 10 years with untreated dental caries demonstrated more trouble sleeping, compromising memory consolidation and learning. Piovesan et al.^12^ revealed that children with advanced dental caries were more likely to report trouble sleeping. Consequently, the treatment of these lesions should be prioritized, aiming not only at reestablishing the esthetic and mechanical functions but also at allowing these patients to perform their daily activities.

The PUFA/pufa index is an excellent indicator of the severity of oral health neglect.^22^ In the present study, the clinical consequences of untreated dental caries in both deciduous and permanent teeth were associated with trouble sleeping due to dental problems among schoolchildren. Mota-Veloso et al.^11^ also found that the clinical consequences of untreated caries may have a negative impact on functional limitations, such as trouble sleeping, in children aged 8-10 years. These results emphasize the importance of immediate care for the consequences of dental caries in children and represent a crucial indicator for the establishment of priorities in public health services and for clinical decision-making.^11^

Contrary to previous studies,^23^ ^,^ ^24^ this research found no association between trouble sleeping due to dental problems and prevalence of toothache. Nevertheless, pain can be defined as an unpleasant experience, interfering with the individual's behavior and activities.^21^ Also, evidence has shown that pain may have a two-dimensional relationship with poor sleep quality, as pain may have a negative influence on sleep duration and inadequate sleep may increase pain sensitivity.^25^ ^,^ ^26^

TDI was not associated with trouble sleeping due to dental problems in the present study. This result could be explained by the low prevalence of schoolchildren with TDI. Moreover, previous reports indicated that TDI did not have a negative impact on the quality of life of children aged 8 to 10 years, which includes sleep-related problems.^27^ Small fractures and some minor trauma might not cause postoperative pain and were not mentioned by the children and their parents; therefore, they might not affect children’s sleep patterns.^28^

Dental caries prevention and the proper treatment of this condition goes beyond the functional and esthetic recovery of teeth. Oral health transcends the oral environment, influencing the general health of the individual.^28^ Evidence has also shown that oral conditions during childhood can have repercussions in adulthood.^29^ Considering this information, we emphasize the importance of periodic visits to the dentist and treatment of oral conditions in children, given their short- and long-term consequences, which include trouble sleeping.

Some limitations of the present study should be discussed. The cross-sectional study design did not allow us to estimate the causality between the explanatory variables and the outcome. Thus, long-term longitudinal studies are necessary to estimate better the association between these oral conditions and trouble sleeping. Also, the evaluation of trouble sleeping was not based on a specific instrument to assess sleep disorders. However, to the best of our knowledge, there was no validated assessment tool for this purpose in Brazil at the time of data collection. Furthermore, the present study included only children from public schools; therefore, inferences should be made with caution.

In conclusion, the findings of the present study suggest that self-reported trouble sleeping due to dental problems is associated with untreated dental caries and its clinical consequences in schoolchildren aged 8 to 10 years, and its prevalence is in agreement with that found in the literature. Thereby, since sleep has a significant impact on the development of children and can be affected by oral conditions, pediatric dentists and other health professionals should instruct parents and guardians on the importance of preventing and treating these conditions to try to improve children’s quality of sleep.

## References

[1] Gruber R, Carrey N, Weiss K, Frappier JY, Rourke L, Broulliette RT (2014). Position statement on pediatric sleep for
psychiatrists. J Can Acad Child Adolesc Psychiatry.

[2] National Sleep Foundation. Sleep in America poll 2006 - teens and
sleep (2015). Sleep Health.

[3] Spruyt K, O'Brien LM, Cluydts R, Verleye GB, Ferri R (2005). Odds, prevalence and predictors of sleep problems in school-age
normal children. J Sleep Res.

[4] Buckhalt JA (2011). Insufficient sleep and the socioeconomic status achievement
gap. Child Dev Perspect.

[5] El-Sheikh M, Buckhalt JA, Cummings M, Keller P (2007). Sleep disruptions and emotional insecurity are pathways of risk
for children. J Child Psychol Psychiatry.

[6] Finan PH, Goodin BR, Smith MT (2013). The association of sleep and pain: an update and a path
forward. J Pain.

[7] Gomes MC, Pinto-Sarmento TC, Costa EM, Martins CC, Granville-Garcia AF, Paiva SM (2014). Impact of oral health conditions on the quality of life of
preschool children and their families: a cross-sectional
study. Health Qual Life Outcomes.

[8] Souza JG, Souza SE, Noronha MS, Ferreira EF, Martins AM (2018). Impact of untreated dental caries on the daily activities of
children. J Public Health Dent.

[9] de Sousa Barbosa T, Gaviao MB, Castelo PM, Leme MS (2016). Factors associated with oral health-related quality of life in
children and preadolescents: a cross-sectional study. Oral Health Prev Dent.

[10] Lima SL, Santana CC, Paschoal MA, Paiva SM, Ferreira MC (2018). Impact of untreated dental caries on the quality of life of
Brazilian children: population-based study. Int J Paediat.

[11] Mota-Veloso I, Soares ME, Alencar BM, Marques LS, Ramos-Jorge ML, Ramos-Jorge J (2016). Impact of untreated dental caries and its clinical consequences
on the oral health-related quality of life of schoolchildren aged 8-10
years. Qual Life Res.

[12] Piovesan C, Antunes JL, Guedes RS, Ardenghi TM (2010). Impact of socioeconomic and clinical factors on child oral
health-related quality of life (COHRQoL). Qual Life Res.

[13] Barbosa TS, Vicentin MD, Gavião MB (2011). Quality of life and oral health in children - Part I: Brazilian
version of the Child Perceptions Questionnaire 8-10. Ciênc Saúde Coletiva.

[14] Martins MT, Sardenberg F, Vale MP, Paiva SM, Pordeus IA (2015). Dental caries and social factors: impact on quality of life in
Brazilian children. Braz Oral Res.

[15] World Health Organization (1997). Oral Health Surveys: basic methods.

[16] Monse B, Heinrich-Weltzien R, Benzian H, Holmgren C, van Palenstein Helderman W (2010). PUFA - an index of clinical consequences of untreated dental
caries. Community Dent Oral Epidemiol.

[17] Andreasen JO, Andreasen FM (1994). Textbook and color atlas of traumatic injuries to the teeth.

[18] Gregory AM, Sadeh A (2012). Sleep, emotional and behavioral difficulties in children and
adolescents. Sleep Med Rev.

[19] Waxmonsky JG, Mayles SD, Calhoun SL, Fernandez-Mendoza J, Waschbusch DA, Bendixsen BH (2017). The Association between disruptive mood dysregulation disorder
symptoms and sleep problems in children with and without
ADHD. Sleep Med.

[20] Dewald JF, Meijer AM, Oort JF, Kerkhof GA, Bögels SM (2010). The influence of sleep quality, sleep duration and sleepiness on
school performance in children and adolescents: a meta-analytic
review. Sleep Med Rev.

[21] Sivertsen B, Glozier N, Harvey AG, Hysing M (2015). Academic performance in adolescents with delayed sleep
phase. Sleep Med.

[22] Kim E, Grover LM, Bortolotti D, Green TL (2010). Growth hormone rescues hippocampal synaptic function after sleep
deprivation. Am J Physiol Regul Integr Comp Physiol.

[23] Gilchrist F, Marshman Z, Deery C, Rodd HD (2015). The impact of dental caries on children and young people: what
they have to say?. Int J Paediatr Dent.

[24] Guskuma RC, Lages VA, Hafner MB, Rando-Meirelles MP, Cypriano S, Sousa ML (2017). Factors associated with the prevalence and intensity of dental
pain in children in the municipalities of the Campinas region, São
Paulo. Rev Paul Pediatr.

[25] Raymond I, Nielsen TA, Lavigne G, Manzini C, Choinière M (2001). Quality of sleep and its daily relationship to pain intensity in
hospitalized adult burn patients. Pain.

[26] Simonelli G, Mantua J, Gad M, St Pierre M, Moore L, Yarnell AM (2019). Sleep extension reduces pain sensitivity. Sleep Med.

[27] Martins MT, Sardenberg F, Bendo CB, Vale MP, Paiva SM, Pordeus IA (2018). Dental caries are more likely to impact on children’s quality of
life than malocclusion or traumatic dental injuries. Eur J Paediat Dent.

[28] Dimaisip-Nabuab J, Duijster D, Benzian H, Heinrich-Weltzien R, Homsavath A, Monse B (2018). Nutritional status, dental caries and tooth eruption in children:
a longitudinal study in Cambodia, Indonesia and Lao PDR. BMC Pediatr.

[29] Broadbent JM, Zeng J, Page LA, Baker SR, Ramrakha S, Thomson WM (2016). Oral health-related beliefs, behaviors, and outcomes through the
life course. J Dent Res.

